# Enzyme immunoassays for the detection of mycotoxins in plant-based milk alternatives: pitfalls and limitations

**DOI:** 10.1007/s12550-022-00467-x

**Published:** 2022-09-02

**Authors:** Christina Rehagel, Ronald Maul, Kim Lara Gützkow, Ömer Akineden

**Affiliations:** 1 Dairy Sciences, Institute of Veterinary Food Science, Justus-Liebig-University Giessen, Ludwigstrasse 21, 35390 Giessen, Germany; 2 Department Safety and Quality of Milk and Fish Products, Max Rubner-Institut, Federal Research Institute of Nutrition and Food, Hermann-Weigmann-Strasse 1, 24103 Kiel, Germany

**Keywords:** Mycotoxins, Plant-based milk alternatives, Immunoassay, Matrix interferences

## Abstract

Plant-based milk alternatives (PBMAs) are a potential source of mycotoxin uptake. To ensure food safety, simple and rapid testing methods of PBMAs for mycotoxins are therefore required. This study investigated the applicability of enzyme immunoassay (EIA) methods for direct testing of PBMAs without sample extraction. Mycotoxin analyses included aflatoxin B_1_ (AFB_1_), sterigmatocystin (STC), ochratoxin A (OTA), deoxynivalenol (DON), and T-2/HT-2-toxin (T-2/HT-2). It was found that the PBMA matrix negatively affected the EIA to varying degrees, thus affecting the reliability of the results. A dilution of PBMAs of at least 1:8 was necessary to overcome matrix interference. This resulted in calculated detection limits of 0.4 µg/L (AFB_1_), 2 µg/L (STC), 0.08 µg/L (OTA), 16 µg/L (DON), and 0.4 µg/L (T-2/HT-2). After analysis of 54 PBMA products from German retail stores, positive results in at least one test system were obtained for 23 samples. However, most positive results were near the calculated detection limit. Control analyses of selected samples by LC–MS/MS for AFB_1_, STC, and OTA qualitatively confirmed the presence of trace amounts of STC in some samples, but quantitative agreement was poor. It was concluded that the high diversity of ingredients used in PBMAs led to a highly variable degree of sample matrix interference even in a 1:8 dilution. Since the use of higher dilutions conflicts with the need to achieve low detection limits, the application of EIA for routine mycotoxin analysis in PBMA for mycotoxins requires further study on the development of a feasible sample preparation method.

## Introduction

The consumption of plant-based milk alternatives (PBMAs) has increased in Germany and other industrialised countries around the world in recent years. In addition to being “vegan”, these products are commonly advertised with claims regarding health, animal welfare, and sustainable agriculture. (Janssen et al. 2016). Persistence Market Research (PMR) reported that the global market for PBMAs is currently estimated at US$ 12.1 billion and is expected to reach US$ 29.5 billion by 2031, growing with a compound annual growth rate of 9.5% (PMR 2021). In 2020, the revenue for PBMAs in Germany was US$ 452 million, which corresponds to a total consumption of around 250 million km (Statista 2021). Further forecasts showed that German consumption of PBMAs will increase to nearly 535 million km by 2026 (Statista 2021). Considering this rapidly increasing consumption, it is of great importance to ensure the food safety of these products. However, PBMAs are not specifically addressed by European Union regulation (EC) No. 1881/2006 which lays down maximum levels (MLs) for mycotoxins (EC 2006).

PBMAs presently available from the German market are an aqueous slurry of various plant materials; the main ingredients are cereals, pseudo cereals, legumes, nuts and seeds, but some also contain sugar, cocoa, or edible oil. Some products additionally contain additives (stabilisers, emulsifiers) and flavours (McClements et al. 2019; Sethi et al. 2016).

In addition to control of raw materials, a rapid and sensitive system of analysis for finished PBMAs is required to verify the safety of such products. Published surveys on contaminants in PBMAs in general are scarce. All contaminants typical for the raw products, i.e. heavy metals or environmental and natural toxins in general, need to be considered also for PBMAs. So far, there are only a few published studies, all with very limited sample size, which report investigations of PBMAs for mycotoxins. Although no study specific for the German market exists, data from other European countries clearly demonstrate that PBMAs may be contaminated by multiple mycotoxins belonging to different chemical groups including trichothecenes and aflatoxins (Arroyo-Manzanares et al. 2019; Hamed et al. 2017, 2019; Juan et al. 2022; Miró-Abella et al. 2017).

Published studies on the occurrence of mycotoxins in PBMAs exclusively utilised liquid chromatography coupled with either tandem mass spectrometry or fluorescence detection. While these methods are convenient in a laboratory environment, they are less suitable for rapid on-site quality control at the production site. For liquid food materials, EIA appear to be a suitable tool for rapid on-site testing and have been used for decades for the analysis of aflatoxin M_1_ in cow’s milk (Pecorelli et al. 2020). Therefore, this study aimed at exploring the possibility to employ a set of in-house EIA methods for different mycotoxins, analysing PBMAs directly without any sample preparation.

## Materials and methods

### Chemicals and reagents

Mycotoxin standards of OTA, AFB_1_, STC, and DON were obtained from Sigma-Aldrich (Taufkirchen, Germany); T-2 toxin was from Biopure (Tulln, Austria). After dissolving the mycotoxin standards in methanol (OTA, AFB_1_, DON, T-2) or acetonitrile (STC), the concentration and purity of all stock solutions (except T-2) were checked by UV spectroscopy (Shimadzu, Duisburg, Germany), using published data (Cole and Schweikert 2003; Cole et al. 2003) for comparison and calculation. ^13^C-labelled standard solutions for AFB_1_, STC, and OTA were obtained from Biopure™ (Romer Labs Deutschland GmbH, Butzbach, Germany). All other reagents and chemicals used were at least of analytical grade. Methanol (LC–MS grade) and acetonitrile (ACN) (LC–MS grade) were purchased from Supelco® (Merck KGaA, Darmstadt, Germany). n-Heptane, dimethylsulfoxide (DMSO), ACN (HPLC grade), and anhydrous magnesium sulphate (MgSO_4_) were purchased from Carl Roth GmbH + Co. KG (Karlsruhe, Germany), while ammonium formate (NH_4_COOH) and acetic acid (HAc) were from Merck KGaA (Darmstadt, Germany). Formic acid (FA) was from VWR International GmbH (Darmstadt, Germany). Ultrapure water was obtained through the use of a water purification device (PURELAB flex 3, ELGA LabWater, Veolia Water Technologies Deutschland GmbH, Celle Germany). AflaTest _WB_ SR^+^ immunoaffinity columns (IAC) were purchased from VICAM (Milford, USA) and contained monoclonal antibodies that specifically bind aflatoxins (B_1_, B_2_, G_1_, G_2_, M_1_, M_2_) and STC. According to the manufacturer, the column capacity was 1000 ng for total aflatoxins and recovery for B_1_, B_2_, G_1_, and G_2_ was ≥ 90% for spiking level of 2 ng and 500 ng.

### Sample materials

A total of 54 samples of various PBMA products, with the majority (*n* = 34) of these labelled as of “organic produce”, were purchased from retail shops and specialised “organic food” stores in the area of Giessen, Hesse, Germany, in 2020. All products were purchased as offered, in original packaging. According to product information, the products originated from 17 different manufacturers from eight countries in Europe; the majority was from German producers (*n* = 28). The main ingredients were water and vegetable raw materials of a content ranging from 8.7 to 17% for cereal-based or pseudocereal-based PBMA (oat, rice, spelt, millet, buckwheat), 2.3–8.4% for nut-based products (hazelnut, almond, coconut, cashew), 4–10% for products based on legumes (soy bean, pea, lupine), 3% for hemp-based products, and 4.9–21% for PBMA consisting of ingredient mixtures (oat + almond, rice + almond, rice + coconut, rice + coconut + cashew). In addition, a few products contained minor amounts of sunflower or rapeseed oil, cocoa, sugar, and salt. Most of the PBMAs without claims of organic produce contained stabilisers and emulsifiers. All products were heat-treated, mostly by ultra-high temperature treatment (> 135 °C); a few were pasteurised. The remaining shelf life of ultra-high temperature-treated PBMA products was > 4 months and for pasteurised PBMA products > 2 weeks at the time of purchase.

### EIA analysis

#### Sample pretreatment

Before opening, each package of PBMA was manually shaken to mobilise sedimented particles. Then, a portion for follow-up analyses of about 50 mL was transferred into plastic test tubes and frozen at − 18 °C. Material from products containing stabilisers or emulsifiers was centrifuged (3000 × g, 10 min, 20 °C). Then, sample material was diluted with EIA buffer solution as required for each test system, and dilutions ranging from 1:2 to 1:20. For AFB_1_, T-2/HT-2, and STC analysis, sample dilutions and toxin standard curves were prepared in phosphate buffered saline (PBS; 0.01 mol/L; pH 7.2) containing 10% methanol. For DON analysis, samples were diluted in PBS (pH 7.2). For OTA analysis, samples were diluted with aqueous NaHCO_3_ solution (0.13 mol/L).

#### Effect of sample matrix on EIA standard curve

Since no certified toxin-negative PBMA material was available, the extent of sample matrix interference was assessed by comparing toxin standard curves made in EIA buffer solution with toxin standard curves made with diluted PBMA. For this series of experiments, one sample each from every major product group was selected. The minimal dilution which yielded standard being congruent with the buffer solution standard curve was then used for analyses of the remaining sample materials.

#### Analysis of artificially contaminated sample material

As an additional quality control, six PBMA materials were artificially contaminated with the mycotoxins under study by adding 20–100 µL of toxin standard solution per millilitre of sample at appropriate concentrations (OTA 0.2–0.8 µg/L; AFB_1_, T-2/HT-2 1–4 µg/L; STC 4–16 µg/L; DON 30–120 µg/L). Four replicates of all standard and sample solutions were analysed, and each PBMA sample was analysed in a single dilution.

#### EIA test procedure

For mycotoxin analysis of PBMA samples, EIA were performed using microtiter plates (MaxiSorp, Nunc, Roskilde, Denmark) as described earlier for AFB_1_ (Gathumbi et al. 2001), STC (Wegner et al. 2016), OTA (Schneider et al. 2001), DON (Curtui et al. 2003), and T-2/HT-2 (Esgin et al. 1989). All EIA were performed based on competitive direct test format, using the double antibody method for DON and T-2/HT-2. The EIA absorbance values at 450 nm were measured using a microplate reader (Tecan Sunrise, Crailsheim, Germany) and evaluated by Magellan calculation software (Tecan, Crailsheim, Germany). EIA absorbance values of standard concentrations were normalised by dividing the mean absorbance values of the standard or diluted sample solution (B) by the absorbance value of the blank (B_0_) and then multiplying by 100 (B/B_0_ × 100).

### Control analyses for AFB_1_, STC, and OTA by LC–MS/MS

#### Sample pretreatment

For the control analyses, five samples which had yielded highly positive results in EIA were selected for LC–MS/MS analysis for AFB_1_, STC, and OTA. Four of these samples (MA11, MA25, MA31, MA54) contained cocoa in addition to their main ingredient, and one sample contained black rice (MA48). For these series of experiments, extracts for AFB_1_ and STC analyses were prepared by liquid–liquid partitioning (LLP) of a 10-mL test portion twice with each 40 mL of ethyl acetate. The two organic phases from each sample were collected and combined, the solvent removed in a rotary evaporator at 50 °C, then the residue dissolved with 10 mL of methanol. One millilitre of the extract was transferred to a conical flask and evaporated at 50 °C in a rotary evaporator. The residue was dissolved in 2 mL of PBS containing 10% methanol and analysed by EIA. The calculated limit of detection (LOD) in LLP extracts was 0.1 µg/L for AFB_1_ and 0.2 µg/L for STC, respectively.

Further purification of the LLP extracts was done using IAC columns. A 5-mL portion of the LLP extract was diluted with 20 mL PBS (pH 7.2), solid particles removed by centrifugation (3000 × g, 10 min, 20 °C), then the supernatant was filtered through a paper filter. The filtered solution was passed through an IAC column, following the manufacturers’ instructions. Toxins were eluted from the column with two, and consecutively added 1.5-mL portions of methanol. The methanolic eluate was collected in a conical flask and 1 mL was used for LC–MS/MS analysis. The remaining solvent (2 mL) was removed in a rotary evaporator at 50 °C and the residue dissolved with 2 mL of 10% methanol/PBS for EIA analysis. Based on the cut-off value of the EIA standard curves, the calculated LOD for IAC extracts was 0.04 µg/L for AFB_1_ and 0.06 µg/L for STC, respectively.

#### LC–MS/MS analysis

The analysis was performed on a 1290 Infinity II LC system (Agilent Technologies Germany GmbH & Co. KG, Waldbronn, Germany). Analytes were separated on a Gemini reversed phase C18 analytical column, 100 × 3.0 mm, 5.0 µm (Phenomenex^®^, Aschaffenburg, Germany), at an oven temperature of 35 °C, while the injection volume was 4 µL. LC separation was performed using a gradient elution of water (with 0.1% formic acid, 300 mg/L ammonium formate) and methanol (with 0.1% formic acid, 300 mg/L ammonium formate) and a flow rate of 0.5 mL/min. The gradient programme started at 5% organic solvent for 0.8 min, raising to 50% by minute 1.5, to 55% by minute 2.5, to 70% by minute 5.5, to 76 by minute 6.5, and to 95% by minute 15.5. Starting from minute 17.0, the organic percentage reverted to the starting conditions of 5% by minute 17.5 and was kept until the end of the run at 19.5 min. MS detection was conducted using a triple quadrupole MS (QTRAP 6500 + , Sciex Germany GmbH, Darmstadt, Germany) operating in both positive and negative electro spray ionisation (ESI) mode and measuring in multiple reaction mode (MRM) with the following settings: curtain gas 40, collision gas medium, temperature 350 °C, the ± ion spray voltage 4500 V, nebuliser gas flow of 50, heater gas flow of 45, and dwell time varied. The analytical parameters for AFB_1_, STC, and OTA are shown in Table 1.

**Table 1:** Analytical parameters of quantitative determination of the analytes and their isotopically labelled internal standards with the HPLC–MS/MS; ESI ( +) mode; multiple reaction monitoring mode; the second product ion was used as a qualifier for the confirmation of identity for each analyte; for all analytes, compound optimisation with the LC–MS was performed

Analyte	Precursor ion (*m/z*)	Product ions (*m/z*)	DP^a^ (V)	CE^b^ (eV)	CXP^c^ (V)
AFB_1_	313.0	285.0	111	33	16
		241.0	111	49	26
^13^C-Aflatoxin B_1_	330.0	301.0	111	33	16
STC	325.0	310.0	96	33	18
		281.0	96	49	32
^13^C-Sterigmatocystin	343.0	297.0	96	49	32
OTA	404.0	239.1	31	29	14
		357.9	31	19	24
^13^C-Ochratoxin A	424.1	250.1	31	29	14

^a^*DP* declustering potential

^b^*CE* collisions energy

^c^*CXP* collision cell exit potential

For sample preparation, 62 µL of the IS mixture was added to 1 mL of the PBMA sample, and the samples were extracted with 938 µL of ACN containing 0.1% FA. After shaking for 10 min (IKA-VIBRAX VXR, IKA^®^-Werke GmbH & CO. KG, Staufen, Germany), 0.1 g NaCl and 0.4 g MgSO_4_ were added and shaken for another 5 min. The samples were centrifuged at 10,000 × g for 7 min at room temperature (Avanti JXN-30, Beckman Coulter GmbH, Krefeld, Germany). A total of 0.8 mL of supernatant was transferred in a tube and 0.8 mL of n-heptane added. After shaking for 5 min (IKA-VIBRAX_VXR, IKA^®^-Werke GmbH & CO. KG, Staufen, Germany), the n-heptane phase was discarded. ACN phase was transferred into a 2-mL reagent tube containing 100 µL DMSO as keeper solvent and the ACN was evaporated until only the DMSO proportion remained (Concentrator plus, Eppendorf AG, Hamburg, Germany). A total of 200 µL ACN with 0.1% FA were added to the residual liquid and vortexed. After additional sonicating for 10 min (Transsonic 460, Elma Schmidbauer GmbH, Singen, Germany), 300 µL of H_2_O was added and the samples were sonicated again for 10 min and vortexed afterwards. The extracts were filtered using a regenerated cellulose 0.45-µm syringe filter unit (ProSense B.V., Munich, Germany). The LLP and IAC extracts were diluted 1/1 with water before the LC–MS/MS analysis.

## Results and discussion

**Fig. 1:**
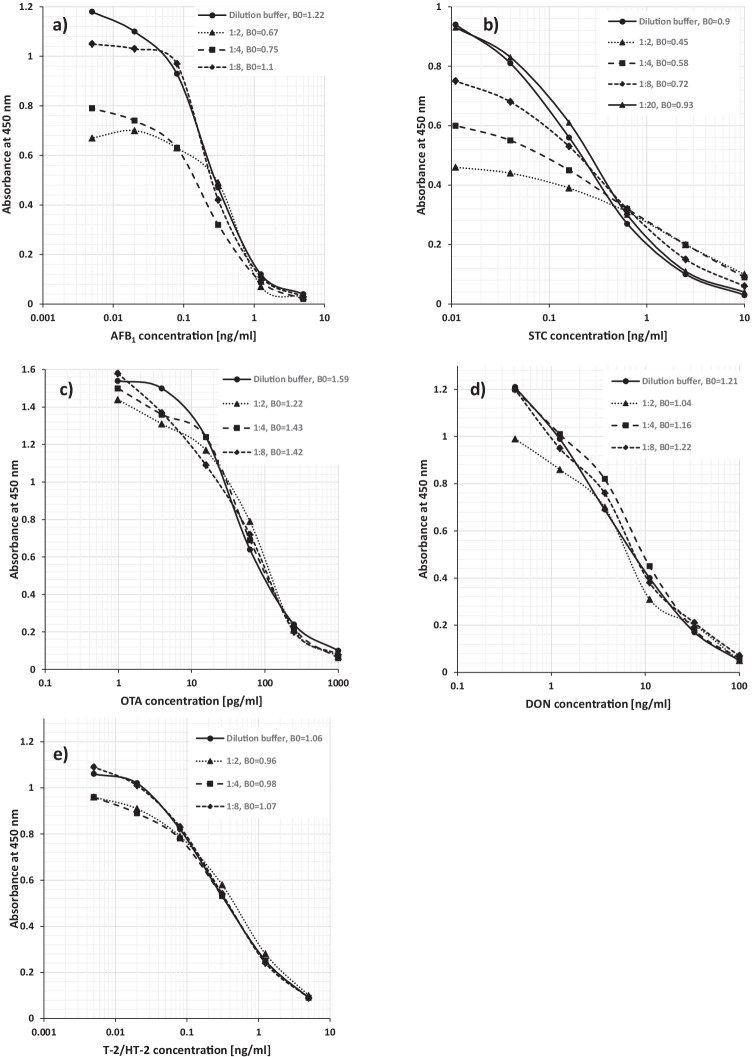
Example of PBMA sample matrix effects on EIA toxin standard curves (only oat-based PBMA shown), indicating the absorbance value of the blank (B_0_) of each standard curve. **a)** AFB_1_: standard curves made in matrix at dilutions with test buffer of 1:2 and 1:4 resulted in a depression of the absorbance at 450 nm. At a dilution of 1:8, the standard curve was almost fully congruent with the standard curve in buffer solution. **b)** STC: strong absorbance signal depression for standard curves made in matrix at dilutions of up to 1:8, at a 1:20 dilution, standard curve which was almost congruent with standard curve in buffer solution. **c)** OTA. **d)** DON. **e)** T-2/HT-2. Each of the six standard curve data points represents the mean absorbance at 450 nm of four replicate wells. The coefficients of variation ranged from 3 to 10% and did not differ between all curves

Given their high sensitivity, EIA seem to be a convenient tool for mycotoxin testing in liquid sample materials such as PBMA. However, it was observed that the highly variable composition of PBMAs and their high content of non-soluble material exerted the matrix influence which did effect each individual test system to varying degrees. This study was also impeded by the fact that no defined reference material, either mycotoxin-free or with certified mycotoxin content, is available for PBMA or comparable matrices. Therefore, we first subjected a larger number (*n* = 54) of PBMA products to EIA analyses at different dilutions with buffer solution. Selected materials from each major group of products were then used to establish toxin standard curves in matrix (Fig. 1). PBMAs up to a dilution of 1:4 yielded strongly left-shifted standard curves with depressed B_0_ values, indicating that false-positives and overestimation of the toxin content were major issues. Except for the STC-EIA, toxin standards prepared in 1:8 diluted PBMA matrix resulted in standard curves which were nearly identical with the buffer solution standard curve, observed for all different matrices. Therefore, a minimum dilution factor of 8 was applied for all subsequent analyses. In the STC-EIA, an even higher dilution (1:20) was required to eliminate left-shifted standard curves. The necessity to dilute PBMAs for EIA analyses negatively affected the achievable, calculated detection limit in sample matrix. The LOD summarised in Table 2 were considered to be still in a relevant concentration range for DON and T-2/HT-2 while for AFB_1_, STC, and OTA, they were probably insufficient.

**Table 2:** Cross-reactions and standard curve parameters of the mycotoxin EIA and calculated detection limit in PBMA

Test system	Known relevant cross-reactions	Standard curve		Minimum sample dilution factor for EIA analysis	Calculated detection limit in PBMA µg/L
		IC_50_ value, mean* ± SD, µg/L	Cut-off value (IC_20_), mean* ± SD, µg/L		
AFB_1_	AFB_1/2_, AFG_1/2_, AFM_1_, AFB2a, AFG2a, AFP_1_, AFQ_1_, Aflatoxicol (Gathumbi et al. 2001)	0.14 ± 0.02	0.05 ± 0.01	8	0.4
STC	*O*-methylsterigmatocystin (Wegner et al. 2016)	0.29 ± 0.09	0.08 ± 0.02	20	2
OTA	OTA, OTB (Schneider et al. 2001)	0.04 ± 0.007	0.01 ± 0.004	8	0.08
DON	DON and its 8-ketotrichothecene analogues (Curtui et al. 2003)	5.15 ± 0.92	1.67 ± 0.48	8	16
T-2/HT-2	T-2, HT-2 (Esgin et al. 1989)	0.23 ± 0.04	0.05 ± 0.01	8	0.4

^*^16–27 plates performed for each test in a period of 12 months, except T-2/HT-2 (5 tests)

Adding toxin standard solution to PBMA material before dilution yielded results which were still quite variable, depending on both type of matrix and spiking level (Table 3). This indicates that even at a 1:8 dilution (STC-EIA: 1:20), some remaining matrix interference could cause up to 50% deviation from the nominal value. A possible reason for these matrix interferences are the proteins contained in PBMAs. For example, Wang et al. (2015) investigated the influence of fish proteins on competitive indirect EIA and demonstrated that fish proteins interfere with immunological reactions by binding to both primary antibodies and enzyme-labelled secondary antibodies.

**Table 3:** EIA results for four different PBMA product groups, artificially contaminated with mycotoxins. For each concentration level, one sample from each category was spiked

		Toxin found, in % of added amount					
Test system	Toxin added, µg/L	Soy	Oat	Almond	Coconut	Mean	RSD^a^, %
AFB_1_	1	80	133	119	83	104	26
	2	90	86	133	128	109	25
	4	97	83	88	156	106	34
STC	4	123	89	148	107	117	25
	8	115	92	142	94	111	23
	16	127	89	142	92	113	26
OTA	0.2	146	85	84	61	94	36
	0.4	114	75	77	69	84	20
	0.8	124	89	91	83	97	18
DON	30	76	104	110	65	89	22
	60	89	133	117	100	110	19
	120	88	129	113	94	106	19
T-2/HT-2	1	64	103	84	63	79	19
	2	79	101	64	66	78	17
	4	87	65	57	56	66	14

^a^Relative standard deviation

When the EIA results of mycotoxin analysis for all 54 samples were grouped according to the main ingredients (Table 4), it became clear that the EIA for DON and T-2/HT-2 in general yielded results which were plausible in view of the trichothecene frequency in cereals. However, with regard to T-2/HT-2, there were two exceptions: one soy-based sample and one hemp-based sample showing a weak positive result for T-2/HT-2. The fact that no studies on the occurrence of mycotoxins in hemp seeds are currently available makes a plausibility assessment difficult in this case. Even though the occurrence of T-2/HT-2 in soy is not common, it cannot be completely excluded. Other study results show that, in addition to cereals susceptible to T-2/HT-2, these toxins can also occur in soybean from Argentina (Barros et al. 2011). With these two exceptions, trichothecene mycotoxins were detected in cereal-containing PBMAs only. The levels measured for these samples corresponded well with contamination data for oats specifically (EFSA 2017; Curtui et al. 2009), and for cereals in general (Gottschalk et al. 2009). Furthermore, they are in good agreement with the results reported by Miró-Abella et al. (2017). Considering that the total amount of solids in these products typically ranged from 5 to 10%, the concentration of these toxins in the cereal ingredients should be about 10–20-fold higher, in a range roughly between 100 and 800 µg/kg for DON, and between 4 and 80 µg/kg for T-2/HT-2. This would be well within the range of reported data for these toxins in European oat.

**Table 4:** EIA results for four categories of PBMA (*n* = 54)

		Soy (*n* = 7)	Almond (*n* = 7)	Oat (*n* = 14)	Single^a^ and mixed^b^ ingredients (*n* = 26)
AFB_1_	*n* positive/*n*	0/7	0/7	2/14	2/26
	Range	-	-	-	0.6–0.8
STC	*n* positive/*n*	1/7	0/7	2/14	0/26
	Range	-	-	-	-
OTA	*n* positive/*n*	1/7	0/7	2/14	1/26
	Range	-	-	0.2–0.4	-
DON	*n* positive/*n*	0/7	0/7	3/14	2/26
	Range	-	-	16–22	17–43
T-2/HT-2	*n* positive/*n*	1/7	0/7	12/14	8/26
	Range	-	-	0.4–4	0.4–1

^a^Spelt, millet, rice, buckwheat, hazelnut, cashew, pea (with/without cocoa), lupin, hemp

^b^Oat + almond, rice + almond, rice + coconut, rice + coconut + cashew

The AFB_1_-EIA showed a positive result for AFB_1_ in one sample based on black whole grain rice, in addition to three pea- or oat-based products with cocoa. On the other hand, almonds are known to potentially contain aflatoxins (Kanik and Kabak 2019), but the AFB_1_-EIA gave negative results for this group of products. The reason for these findings could be that the LOD for this toxin in these matrices did not allow sufficiently sensitive analysis. Assuming that the raw materials complied with European Union regulation 1881/2006, the aflatoxin levels which could be expected in PBMA based on soy or almonds would probably be below 0.4 µg/L, which is the calculated LOD of the AFB_1_-EIA. A similar situation was observed for the STC-EIA (LOD 2 µg/L), which gave positive results in just one soy-based PBMA and in two oat-based products. The few positive results in the OTA-EIA (LOD 0.08 µg/L) for PBMA based on soy or oat were found for the same samples. All these three products contained cocoa, in addition to the main ingredient. Furthermore, a weakly positive result for OTA was found in the sample based on black whole grain rice.

**Fig. 2:**
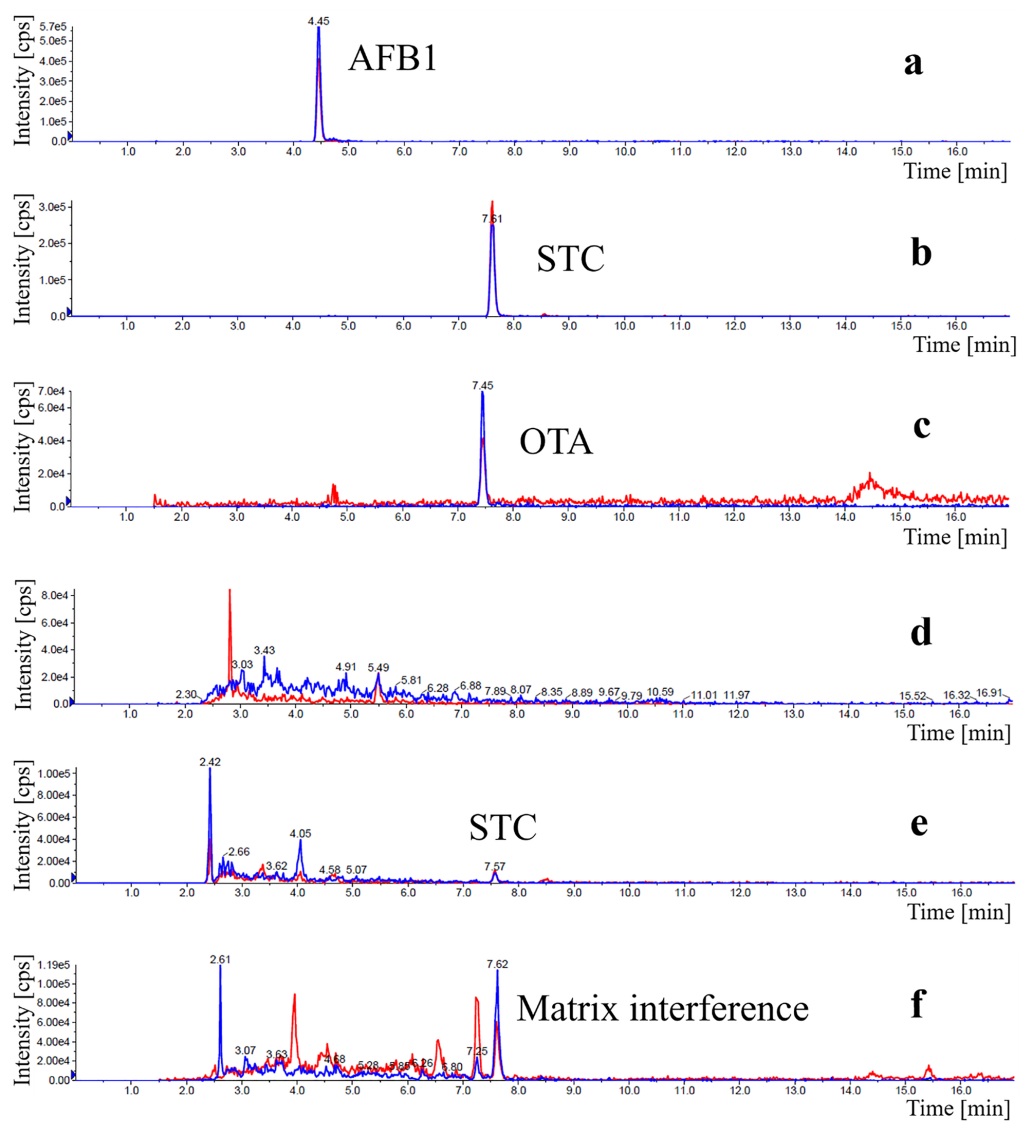
LC–MS/MS chromatograms in ESI ( +) mode of an AFB_1_ **a**, STC **b**, and OTA **c** reference and of a STC positive soy-based PBMA sample (MA11) containing cocoa (**d**–**f**). Extract ion chromatograms in the multiple reaction monitoring mode (MRM) showing mass transitions (m/z) **a**, **d** 313.0 → 285.0; **b**, **e** 325.0 → 310.0; and **c**, **f** 404.0 → 239.1

Further work on elucidation of matrix effects therefore focussed on products containing cocoa as an ingredient and the product based on black whole grain rice. In fact, these products yielded the highest results in the AFB_1_-EIA, STC-EIA, or OTA-EIA. Two products were oat-based, one was based on soy, and another was based on peas, but all contained cocoa according to package labels. Although cocoa is known to be susceptible to aflatoxins and OTA contamination (Copetti et al. 2011; Gilmour and Lindblom 2008; Raters and Matissek 2003), it seemed unlikely that the high levels in the EIA were caused by the low percentage of cocoa (< 1.5%) in the product; for this reason, these samples were additionally analysed by LC–MS/MS. In an initial attempt to improve the detection limit by lowering the sample dilution factor, extracts were prepared by LLP of these samples with ethyl acetate, followed by a further clean-up step on IAC columns. The results of this comparison analysis (Table 5) showed virtually no agreement between EIA and LC–MS/MS. Furthermore, the EIA results for diluted sample and sample extracted by LLP or IAC also gave fully inconsistent results. LLP extracts were still positive, albeit at lower levels, in the tests for AFB_1_ and STC. IAC extracts were all EIA negative for AFB_1_, but still weakly positive for STC. Sample MA54, which had been negative in diluted sample material, were tested positive for STC by EIA in LLP and IAC extracts. This indicates that at least for cocoa-containing samples, the EIA are not applicable to PBMA without significant improvement of the sample preparation method. Further work will study on a broader sample matrix basis, whether similar discrepancies are to be expected for other PBMA products. The costlier and time-consuming LC–MS/MS analysis achieves lower LOQ. Thus, traces of STC were detected in the comparative LC–MS/MS analysis in some samples (Fig. 2), indicating that the further work is warranted to clarify the contamination situation. Although LC–MS/MS analysis revealed the presence of a peak showing both typical mass transitions for OTA in some PBMAs, OTA contamination could not be confirmed. Due to the small retention time shift of 0.2 min compared to the OTA standard, the peak was caused by a matrix interference (Fig. 2). Additionally, an OTA adduct can be out-ruled as the mass spectrum does not show the typical pattern for a chlorine-containing compound (data not shown).

**Table 5:** Comparison of EIA and LC–MS/MS results for AFB_1_, STC, and OTA in diluted sample, in extracts after liquid–liquid partitioning (LLP), and in LLP extracts plus IAC clean-up for five selected PBMA samples

Sample no.	Sample description	AFB_1_, µg/L		STC, µg/L		OTA, µg/L	
		EIA	LC–MS/MS	EIA	LC–MS/MS	EIA	LC–MS/MS
MA11	Sample (soy drink cocoa)	< 0.4	< 0.002	2	< 0.005	0.7	< 0.288
MA11	LLP extract	0.3	< 0.002	2	< 0.002	n.a	< 0.288
MA11	IAC extract	< 0.04	< 0.002	0.09	< 0.002	n.a	< 0.288
MA25	Sample (oat drink cocoa)	0.5	< 0.002	3	< 0.005	0.4	< 0.288
MA25	LLP extract	0.3	< 0.002	2	< 0.002	n.a	< 0.288
MA25	IAC extract	< 0.04	< 0.002	0.08	< 0.002	n.a	< 0.288
MA31	Sample (oat drink cocoa)	0.5	< 0.002	3	< 0.002	0.2	< 0.288
MA31	LLP extract	0.4	< 0.002	2	< 0.002	n.a	< 0.288
MA31	IAC extract	< 0.04	< 0.002	0.07	< 0.002	n.a	< 0.288
MA48	Sample (black whole grain rice)	0.6	< 0.002	< 2	< 0.002	0.1	< 0.288
MA48	LLP extract	0.2	< 0.002	1	< 0.002	n.a	< 0.288
MA48	IAC extract	< 0.04	< 0.002	0.1	< 0.002	n.a	< 0.288
MA54	Sample (pea drink cocoa)	0.8	< 0.002	< 2	0.06	< 0.08	< 0.288
MA54	LLP extract	0.2	< 0.002	2	< 0.002	n.a	< 0.288
MA54	IAC extract	< 0.04	< 0.002	0.2	< 0.002	n.a	< 0.288

*n.a.* not analysed, *LOD* limit of detection (EIA for samples: AFB_1_, 0.4 µg/L; STC, 2 µg/L; OTA, 0.08 µg/L; EIA for LLP extracts: AFB_1_, 0.1 µg/L; STC, 0.2 µg/L; EIA for IAC extracts: AFB_1_, 0.04 µg/L; STC, 0.06 µg/L; LC–MS/MS for sample preparation as described above in LC–MS/MS analysis: AFB_1_, 0.002 µg/L; STC, 0.002 µg/L; OTA, 0.288 µg/L), *LOQ* limit of quantification (LC–MS/MS for sample preparation, calculated for the conventional clean up without LLP or IAC, as described above in LC–MS/MS analysis: AFB_1_, 0.008 µg/L; STC, 0.005 µg/L; OTA, 0.95 µg/L)

Our data suggest that there is the possibility of a mycotoxin contamination in PBMA that can contribute to the overall mycotoxin exposure. This finding might be of interest for consumer groups that consume particularly high amounts of these drinks. However, currently, there are no PBMA consumption data available for Germany. Thus, an estimation of the contribution to the overall exposure is not feasible at this point.

In conclusion, this study showed that the PBMA matrix is highly complex and presents a challenge for EIA methods, although not all test systems were found to be equally susceptible to matrix interference. In any case, careful study of the effectiveness of sample treatment is required for each EIA and should be followed by broad validation studies. Before EIA could be recommended for general routine screening of PBMAs, such studies should include all relevant varieties of composition and all product groups. Unlike milk, analysis of PBMA after dilution with buffer has a high risk of false-positive or false-negative results.
